# Preparation of Discontinuous Cu/SiO_2_ Multilayers—AC Conduction and Determining the Measurement Uncertainty

**DOI:** 10.3390/s23052842

**Published:** 2023-03-06

**Authors:** Aleksandra Wilczyńska, Andrzej Kociubiński, Tomasz N. Kołtunowicz

**Affiliations:** 1 Department of Electrical Devices and High Voltage Technology, Lublin University of Technology, 20-618 Lublin, Poland; 2 Department of Electronic and Information Technologies, Lublin University of Technology, 20-618 Lublin, Poland

**Keywords:** nanocomposites, multilayers, magnetron sputtering, impedance spectroscopy, SEM, measurement uncertainty

## Abstract

This paper presents a test stand for testing alternating current electrical parameters of Cu–SiO_2_ multilayer nanocomposite structures obtained by the dual-source non-reactive magnetron sputtering method (resistance, capacitance, phase shift angle, and dielectric loss angle tangent δ). In order to confirm the dielectric nature of the test structure, measurements in the temperature range from room temperature to 373 K were carried out. The alternating current frequencies in which the measurements were made ranged from 4 Hz to 7.92 MHz. To improve the implementation of measurement processes, a program was written to control the impedance meter in the MATLAB environment. Structural studies by SEM were conducted to determine the effect of annealing on multilayer nanocomposite structures. Based on the static analysis of the 4-point method of measurements, the standard uncertainty of type A was determined, and taking into account the manufacturer’s recommendations regarding the technical specification, the measurement uncertainty of type B.

## 1. Introduction

Among the wide range of nanomaterials and nanostructures, a special place is occupied by metal-dielectric nanocomposites, which include metallic nanoparticles arranged inside a dielectric or ferroelectric matrix [1,2,3]. Reducing the dimensions of composite materials does not change their chemical composition. A characteristic feature of nanocomposite structures is the size of at least one phase, which does not exceed 100 nm. Modifications of the structure and composition of nanocomposites enable the adjustment of their physical properties [4,5]. An important property of the discussed structures is their surface to volume ratio, which is much higher than in the case of macrocomposites. The structure of the nanocomposite determines the type of electric charge transfer mechanism, the knowledge of which is necessary for the selection of potential applications in devices [6,7].

There are two reasons for the great interest of researchers in the translational properties of metal-dielectric nanocomposite materials. On one hand, disordered systems are a difficult field in a purely academic sense. For many years, the theory of charge transport through semiconductors was mainly limited to crystalline systems in which atoms are located at regular nodes of the crystal lattice [8,9]. The concepts often used in textbooks to describe the transport of charge carriers in crystalline semiconductors are based on the assumption of long-range order, so they cannot be applied to electron transport in disordered materials such as nanocomposites. The development of a consistent theory of charge transport in such systems was (and still is) a very difficult task. The fact is that granular metal-dielectric nanocomposites are mixtures of conductive and insulating or weakly conducting nanoparticles. They are most often discussed within the framework of so-called percolation models [10,11]. These models describe the properties of granular systems as a function of the ratio of the content of conductive nanoparticles *x* to the total volume of the system. According to the percolation model, an increase in the content of the metallic phase to *x = x_C_* (percolation threshold) causes a transition from dielectric to metallic conductivity [12,13,14].

A good method of determining the mechanisms taking place in nanocomposites is impedance spectroscopy [15,16]. The principle of dielectric spectroscopy is mainly to determine the frequency dependent combined dielectric permeability. It also provides information on molecular dynamics as well as important material parameters such as static permeability, alternating current electrical conductivity, and dielectric relaxation [17,18]. Almost all materials are dielectrics, that is, they do not exhibit macroscopic DC conductivity, but instead act as capacitance. Dielectric testing is conducted mainly for the following two reasons. First, the acquired data provide detailed information about the electrical properties of the samples. This provides a lot of theoretical information and also has practical application in the electronics industry, particularly, in the development of semiconductor devices as well as in the characterization of insulation materials. Second, the technique serves as an analytical tool by which the obtained dielectric data can be linked to other properties such as changes in the material morphology.

The dielectric properties of nanomaterials have potential applications in capacitors, sensors, and memory devices. The influence of frequency on the dielectric behavior and AC conductivity of nanomaterials provides key information on the phenomena of conduction in these materials [19,20,21,22]. It should be noted that the dielectric properties and electric transport of nanomaterials are very different from the properties of materials on a micro- or macrometric scale.

The structure and morphology of nano-grained metal-dielectric composites determine their extraordinary electrical properties, the research of which allows us to broaden the knowledge about the mechanisms of carrier transport in a non-homogeneous medium at the nanoscale [23,24]. From a practical point of view, it is important to study their conductivity properties at different temperatures. In order to describe the electrical properties of nanocomposites, several conduction mechanisms have been proposed, which are realized in various temperature ranges and depend on the material of the nanoparticles and the presence of additional oxides around the nanoparticles of the metallic phase [25,26,27,28,29].

Granular metal-dielectric nanocomposites, in which the matrix is SiO_2_, are of great interest due to their extraordinary optical [30,31] and electro-physical properties [32,33]. In particular, they provide the possibility of fine-tuning the optical response and electrical resistance on a large scale, and non-magnetic Cu*_x_*(SiO_2_)_(100−*x*)_ nanocomposites show a gigantic Hall effect [34,35], are characterized by a hopping of electron transport [36,37], and exhibit the phenomenon of coinless inductance [38,39]. Therefore, understanding the structure of granular nanocomposites and developing an optimal method of their preparation is a necessary condition to control their properties. Depending on the content of the metallic phase *x* in the dielectric matrix, there are three types of electrical conductivity in the granular nanocomposites.

The temperature dependence of the electrical conductivity of nanocomposites in the dielectric range within the low temperature range mostly shows that there is a characteristic variable-range hopping conductivity. In the transition range (*x ≈ x_C_*), some of the metallic grains fuse together to form a labyrinthine structure that, with a decrease in metallic phase content, gradually disintegrates into isolated nanoparticles dispersed in a non-conducting matrix. The electrical conductivity in this case is due to percolation along the metallic junctions and electron tunneling between the insulated particles. Then, the contribution of electrical conductivity due to thermally activated tunneling becomes comparable to the contribution due to percolation, and the temperature coefficient of resistivity becomes negative. When the metal content is above the percolation threshold *x_c_*, it is said to be a metallic region in which the temperature coefficient of resistance is positive [40,41].

The best adapted and clean techniques for obtaining nanoparticles in a dielectric matrix are ion-beam sputtering and magnetron sputtering [2,42,43]. In this way, it is possible to obtain nanocomposites with a specific phase composition, showing the phenomenon of electron tunneling, hopping of electrons, or coinless inductance [1,6,44].

The aim of this work was to develop a technological process for the preparation of a metal-dielectric nanocomposite with the composition of Cu/SiO_2_ and to study its basic AC parameters. Additionally, uncertainties of A and B type measurements were estimated. The tests were carried out directly after the preparation process and after annealing.

## 2. Technology of Obtaining Multilayer Nanocomposite Structures and Measurement Method

**Figure 1:**
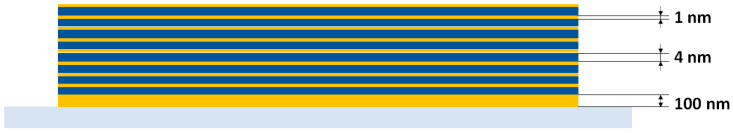
Scheme and dimensions of the structure in the current to plane configuration.

The process of obtaining Cu-SiO_2_ nanocomposites began with the preparation of glass substrates, on which, after cleaning in an ultrasonic cleaner, Kapton masks were applied, enabling a rectangular structure with dimensions of 15 mm × 5 mm to be obtained. After the structures were mounted on the turntable, they were placed in the vacuum chamber of a NANO 36^TM^ (Kurt J. Lesker Company, Dresden, Germany). The sputtering process was initiated in a vacuum of 10^−7^ Torr, 75 sccm argon atmosphere, and 100 W plasma power for the metal material source and 800 sccm, 59 W for the dielectric material. The material sources were alternately sputtered by non-reactive magnetron sputtering in the presence of argon. The first layer was 100 nm of copper, then 4 nm of SiO_2_ and 1 nm of copper layers were alternately deposited eight times. After the structures had been removed from the sputtering machine, the Kapton mask was removed and silver paste contacts were applied. The diagram of the obtained structure is shown in Figure 1.

**Figure 2:**
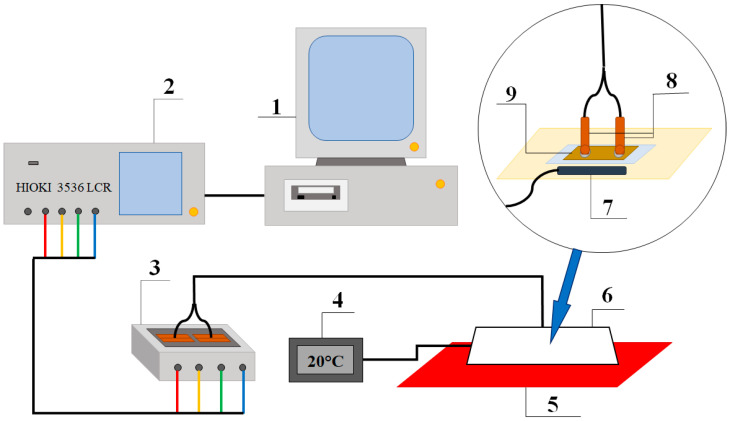
Stand for testing the AC properties of granular nanocomposites in the frequency range from 4 Hz to 8 MHz and in the temperature range from room temperature to 393 K, consisting of: 1—PC running the MATLAB program, 2—impedance meter HIOKI 3536, 3—4-point HIOKI 9261 test fixture relay, 4—temperature display, 5—heating table, 6—Styrofoam housing to maintain the temperature in the test sample environment, 7—temperature sensor, 8—measuring contacts, 9—tested sample.

In order to test the electrical properties of the obtained samples, they were measured using the 4-point method with a HIOKI 3536 impedance meter (Hioki E.E. Corporation, Nagano, Japan) in the frequency range from 4 Hz to 8 MHz. Due to the high resistance of the obtained structures, statistical measurements of all parameters obtainable with the impedance meter were carried out. In order to streamline the process, a program was written in the MATLAB environment, forcing the simultaneous measurement of 17 parameters. Confirmation of the dielectric nature of the studied structures required the construction of a stand that would enable experiments at elevated temperatures. Figure 2 shows the measuring equipment that allows for the testing of AC properties at a controlled temperature. The experiments were performed from 303 K to 393 K on the nanocomposite immediately after spraying, and then annealed at 473 K in the air atmosphere.

## 3. Result

**Figure 3:**
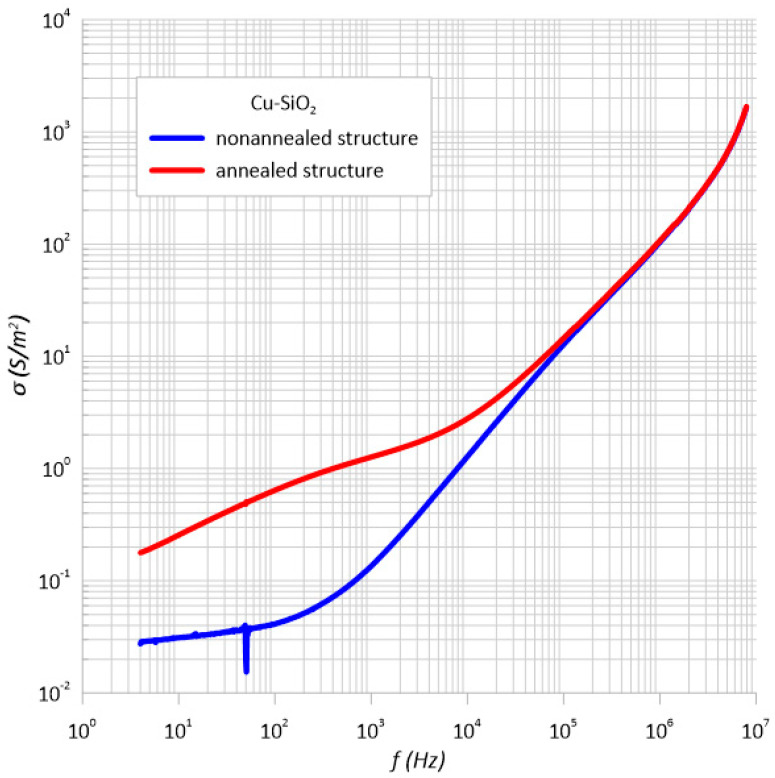
Frequency dependence of the conductivity of the Cu-SiO_2_ nanocomposite measured at room temperature before and after annealing.

Figure 3 shows the frequency dependence of the Cu-SiO_2_ nanocomposite conductivity measured at room temperature immediately after the preparation process and after heating. In the structure diagram before annealing, it can be seen that in the low frequency range up to approximately 100 Hz, the conductivity practically did not depend on the frequency. As it continued to increase, the conductivity increased by 4-orders of magnitude. Changes of this type are characteristic of the hopping mechanism of transferring charges in the material [25,26]. The hopping model assumes that in a nanocomposite, electrons jump between the nearest neighboring potential wells. These wells are metal nanoparticles embedded in a dielectric matrix. In this case, thanks to the developed method of deposition of discontinuous layers, it was possible to obtain a material with such a structure. The sample heating caused changes in the conductivity-frequency characteristics. Its value also increased with increasing conductivity by 4-orders of magnitude. However, the changes are in two stages. In the range up to approx. 1500 Hz, the slope of the almost rectilinear section of the dependence σ(f) was approximately 0.4, and at higher frequencies, it was 0.8. A slope of 0.8 corresponds to the hopping conductivity by the Mott model [45].

**Figure 4:**
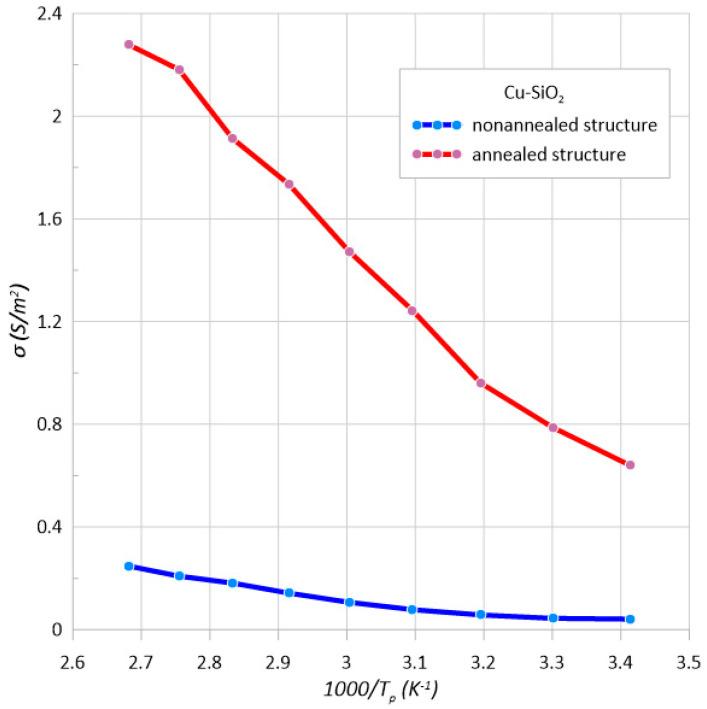
Arrhenius plot of conductivity at the frequency *f* = 100 Hz of the Cu–SiO_2_ nanocomposite before and after annealing at *T*_a_ = 473 K.

Figure 4 shows an Arrhenius plot of conductivity measured at 100 Hz of the structure before and after annealing. In the structure immediately after the preparation the conductivity increases with temperature, the material exhibited a dielectric nature of conductivity. In such a case, the derivative of the conductivity after the temperature dσ/d*T* had positive values [46]. On this basis, we can conclude that the developed method allows one to obtain a Cu–SiO_2_ nanocomposite below the percolation threshold. This means that there are inclusions of metal in the SiO_2_ matrix with no electrical contact between them—so they create potential wells. The thermal activation energy of electrons needed for the jump between the wells was determined and was Δ*E*_1_ ≈ 0.25 eV.

On the basis of the Arrhenius diagram of the structure after heating conductivity determined for the frequency of 100 Hz (Figure 4), the thermal activation energy of the electrons was calculated and was approximately 0.15 eV. The conductivity increased with increasing temperature, which corresponded to the dielectric nature of conductivity.

**Figure 5:**
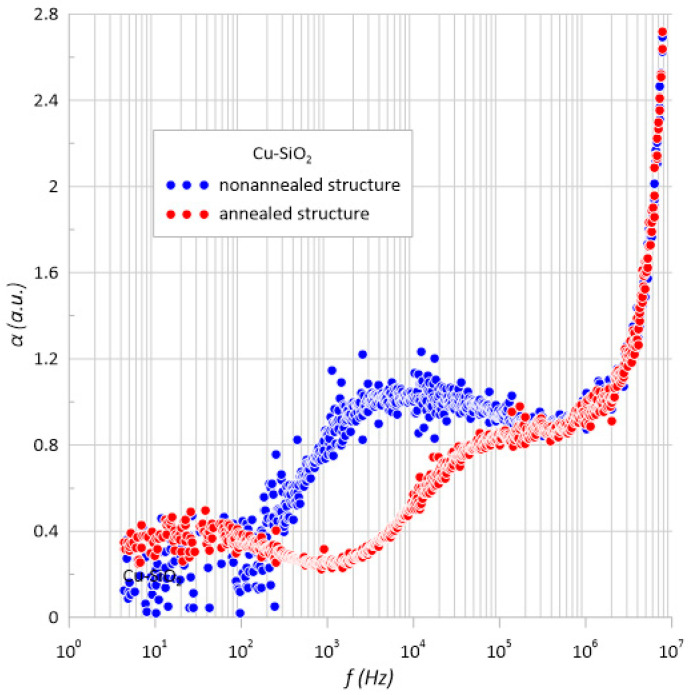
Frequency dependence of the frequency coefficient α of the Cu-SiO_2_ nanocomposite measured at room temperature before and after annealing *T*_a_ = 473 K.

Figure 5 shows the dependence of the frequency permeability coefficient. In the case of the unannealed structure, the maxima can be observed. However, in the case of the annealed structure, a maximum was also visible in the low frequency range, which proves the uniform oxidation of copper nanoparticles. The parabolic shape of the characteristics confirmed the occurrence of a hopping conduction mechanism in the examined structures. The increase in the value of the α coefficient in the frequency range above 10^6^ Hz may be caused by the accumulation of charge on the surface of the structure.

**Figure 6:**
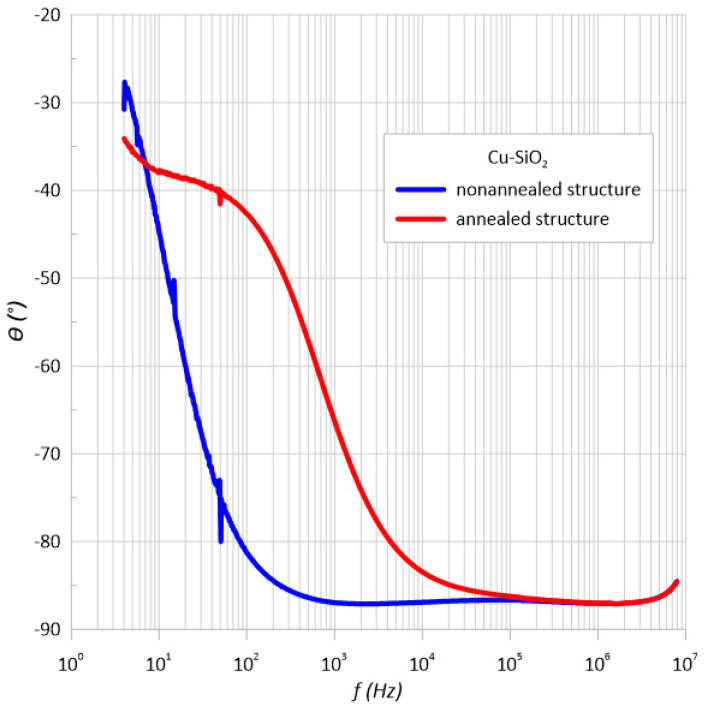
Frequency relationship of the phase shift angle *θ* measured at room temperature immediately after the preparation process and after heating at *T*_a_ = 473 K.

Based on the dependence of the phase shift angle as a function of frequency (Figure 6) for the structure both before and after annealing, it can be concluded that the material has a capacitive character. The phase angle was negative over the entire measuring range. In addition, it can be seen that its value decreased with frequency, reaching almost −90 degrees. This behavior of phase angle with frequency occurred for the conventional parallel RC circuit.

### 3.1. Structural Studies of the Nanocomposite Cu-SiO_2_

**Figure 7:**
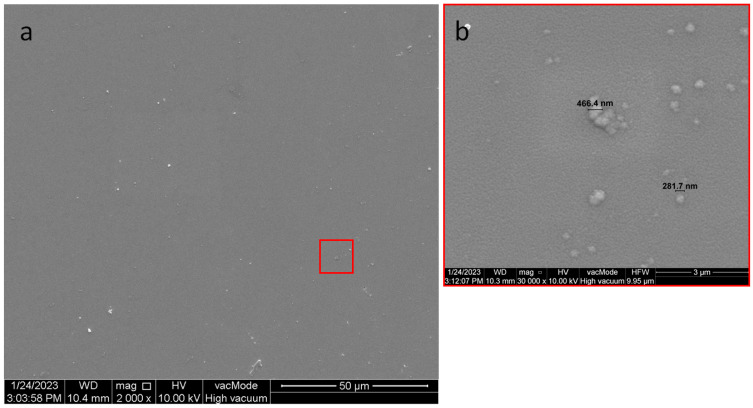
SEM images of the structure before annealing in 50 µm (**a**) and 3 µm (**b**) resolution.

**Figure 8:**
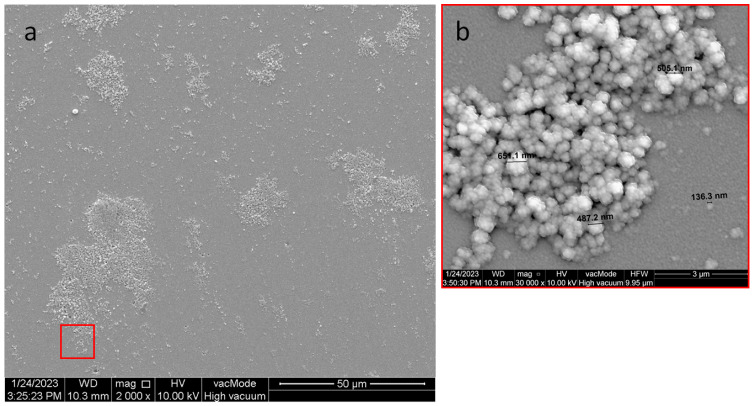
SEM images of the structure after annealing in 50 µm (**a**) and 3 µm (**b**) resolution.

In order to confirm the granular structure of the obtained nanocomposite and to determine the changes induced by annealing, structural studies were carried out with a Quanta^TM^ 250 FEG microscope SEM (FEI Company, Eindhoven, The Netherlands). Annealing in air can affect the oxidation behavior of conductive grains, thereby radically changing their properties. Figure 7 shows a microscopic image of the structure obtained immediately after sputtering. Based on studies, it can be concluded that the annealing process of the structure caused the formation of larger clusters of metal grains, as shown in Figure 8. The merging of grains into clusters may be the reason for the two-step conduction mechanism presented in the previous section.

By comparing the grain sizes in Figure 7 and Figure 8, annealing caused a slight increase in the copper grains. It can be seen that there were very few bright areas in Figure 7. These were copper grains distributed in the SiO_2_ dielectric matrix. On the other hand, in Figure 8, there were many more bright areas, which may mean that they are starting to merge with each other, possibly resulting in exceeding the critical content of the metallic phase, at which well-conductive paths begin to form. This is caused by the process of oxidation of the metal, and thus the increase in the diffusion barrier of the nanoparticle. In the structure before annealing, one of the largest grains had a diameter of about 466.4 nm, while after annealing was 651.1 nm.

### 3.2. Standard Uncertainty of Type a Measurements

Carrying out a series of four statistical measurements made it possible to determine the standard measurement uncertainty of type A. The HIOKI 3536 LCR impedance meter is an automatic device and is burdened with a certain measurement error. Based on the obtained results of the Cu-SiO_2_ granular structure resistance, the statistical evaluation of the standard uncertainty was determined using the A method in accordance with the equation of the standard uncertainty estimator [47].

(1) $$u_{A}\left(x\right)=\sqrt{\frac{1}{\left(N-1\right)N}\overset{N}{\underset{i=1}{\sum }}{\left(x_{i}-\bar{x}\right)}^{2},}$$

where *N* is the number of measurement data; *i* is this measurement; $\bar{x}$ is the mean of the measurements.

**Figure 9:**
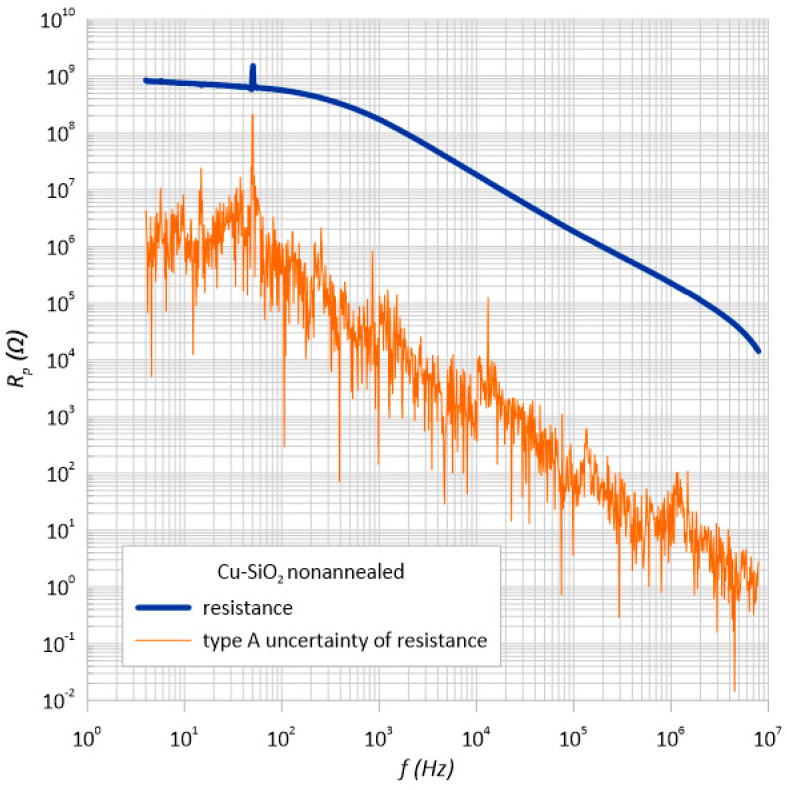
Frequency dependence of the resistance *R*_p_ and its estimator *u*(*R*_p_) measured at room temperature immediately after the preparation process.

**Figure 10:**
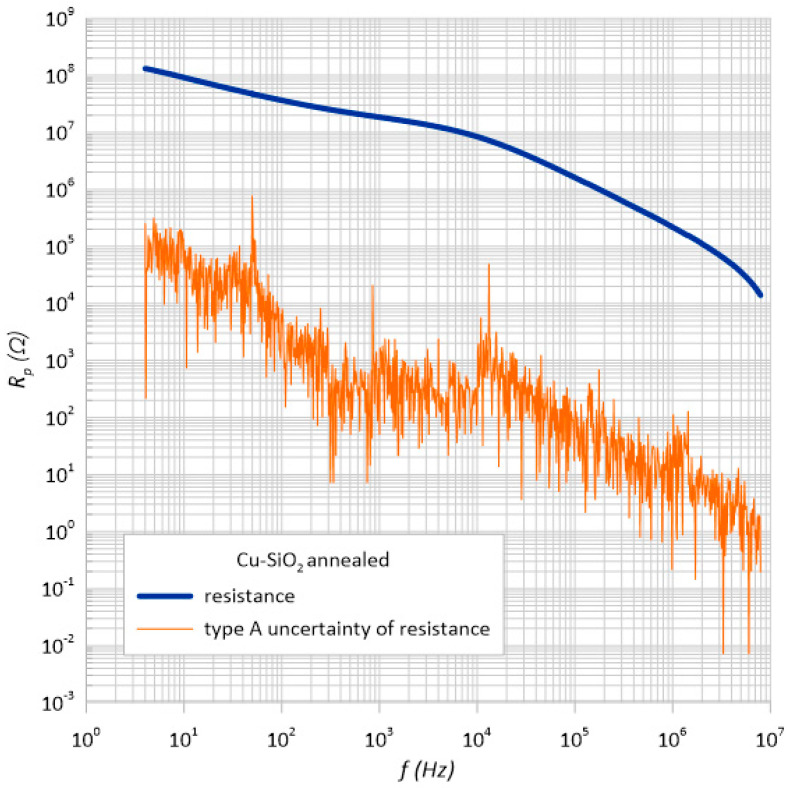
Frequency dependence of the resistance *R*_p_ and its estimator *u*(*R*_p_) measured at room temperature after annealing at *T*_a_ = 473 K.

Figure 9 and Figure 10 present the estimation of the type A uncertainty of the structure before and after heating. For the first case, for the frequency of 10 kHz, the error was approximately 519 Ω, and for the second, it was 119 Ω with such high resistance values of the tested sample constituting 0.003% and 0.0015% of the measurement, respectively.

Table 1 presents the percentage values of errors determined by the *A* method for characteristic measurement points with different frequency values.

### 3.3. Standard Uncertainty of Type B Measurements

The general availability of the specification of the HIOKI 3536 impedance meter enables the calculation of the non-static uncertainty using the *B* method. We estimated it based on the analysis of the properties of the measuring instrument and on the analysis of other sources of error. Usually, in order to determine the type *B* uncertainty, the limit error that is predicted for the operation of a given device is used [43]. An analysis of the standard uncertainty of measurement type *B* for two characteristic parameters given by the producer in the impedance meter specification (i.e., measurement of impedance *Z* and the phase shift angle *θ*). The standard uncertainty is then defined by the equation:

(2) $$\sigma_{s}=\frac{1}{\sqrt{3}}\Delta m_{x}=\frac{m_{x}\delta m_{x}}{\sqrt{3}},$$

where Δ*m_x_* is the maximum error value.

For a value below 100 Ω:

(3) $$\delta m_{x}=\pm \left(A+B\times \left|\frac{range}{m_{x}}\right|-1\right),$$

For a value higher than 1 kΩ:

(4) $$\delta m_{x}=\pm \left(A+B\times \left|\frac{10\times m_{x}}{range}\right|-1\right),$$

where *A*, *B* are the accuracy factors and range is the measuring range.

**Figure 11:**
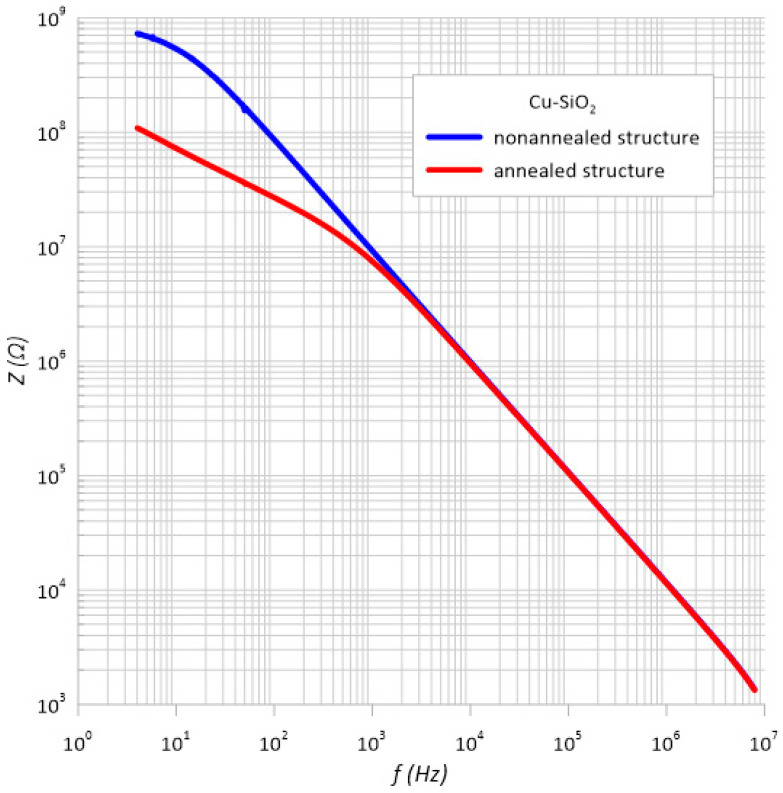
Dependence of impedance on the frequency of an unheated and heated structure.

Figure 11 shows the dependences of impedance as a function of frequency. As can be seen, throughout the frequency range, the impedance values were greater than 1 kΩ. Therefore, only Equation (4) was used to calculate the uncertainty of type *B*.

**Figure 12:**
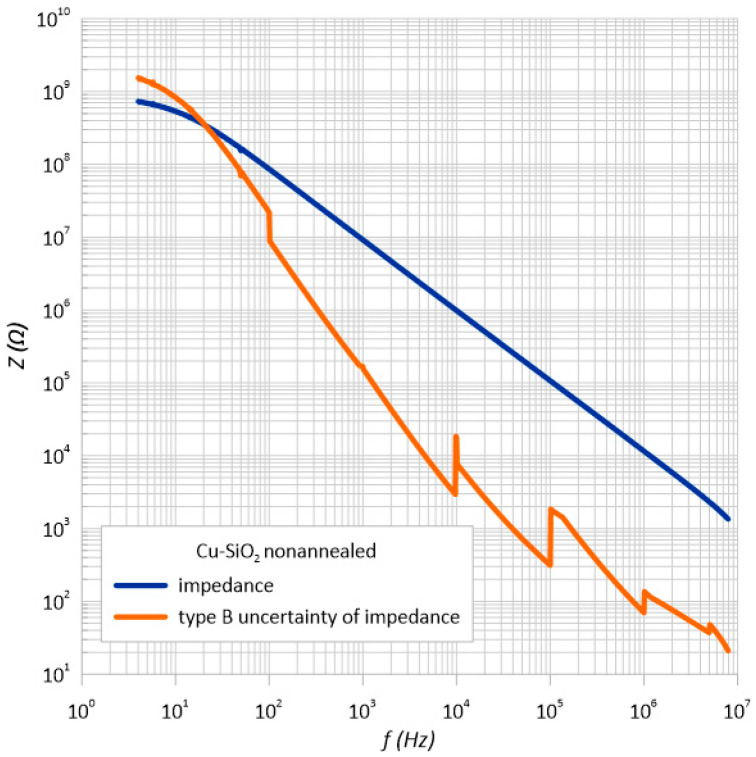
Frequency dependence of the impedance and standard impedance uncertainty of measurement type B *u*(*Z*) of the unheated structure.

**Figure 13:**
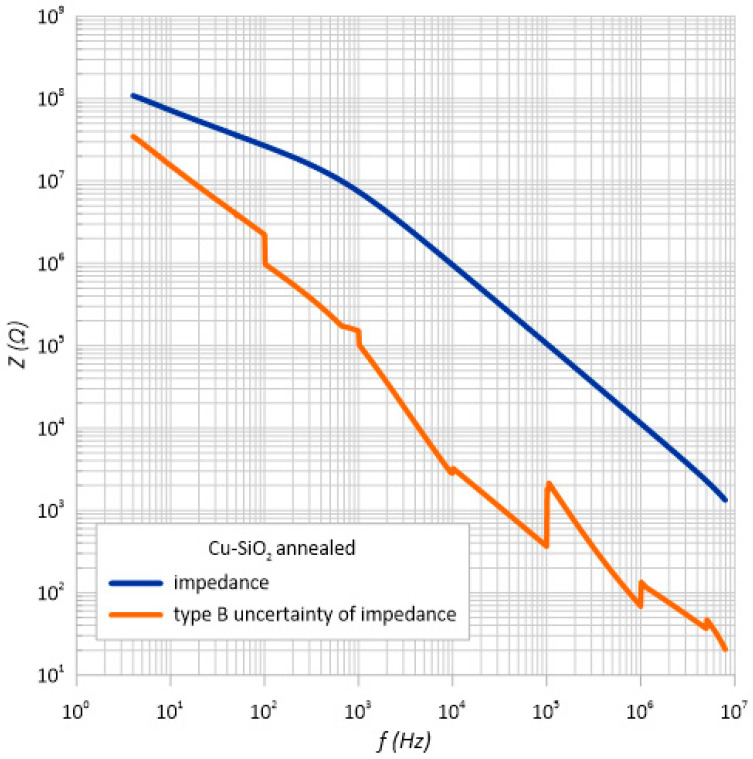
Frequency dependence of the impedance and standard impedance uncertainty of measurement type B *u*(*Z*) of the heated structure.

Taking into account the manufacturer’s recommendations, the standard uncertainty was calculated using the *B* method for the impedance *Z*. The same as for the type *A* uncertainty, the measurement error had a very large value. Figure 12 and Figure 13 show the impedance measurements with type *B* uncertainty.

Table 2 presents the percentage values of errors determined by the *B* method for impedance *Z* measurement points with different frequency values.

**Figure 14:**
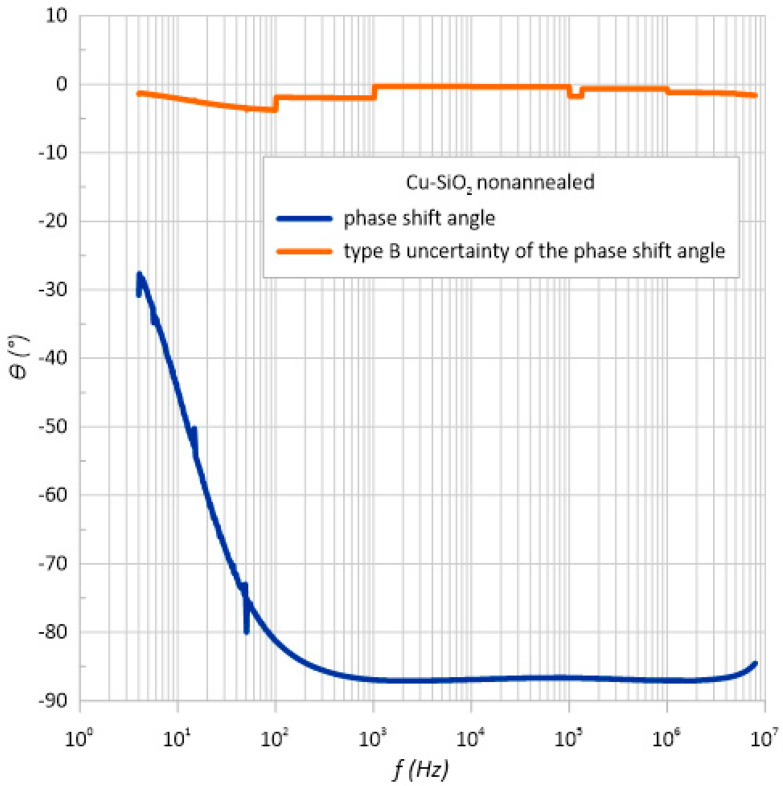
Frequency dependence of the impedance and standard impedance uncertainty of measurement type B u(θ) of the unheated structure.

**Figure 15:**
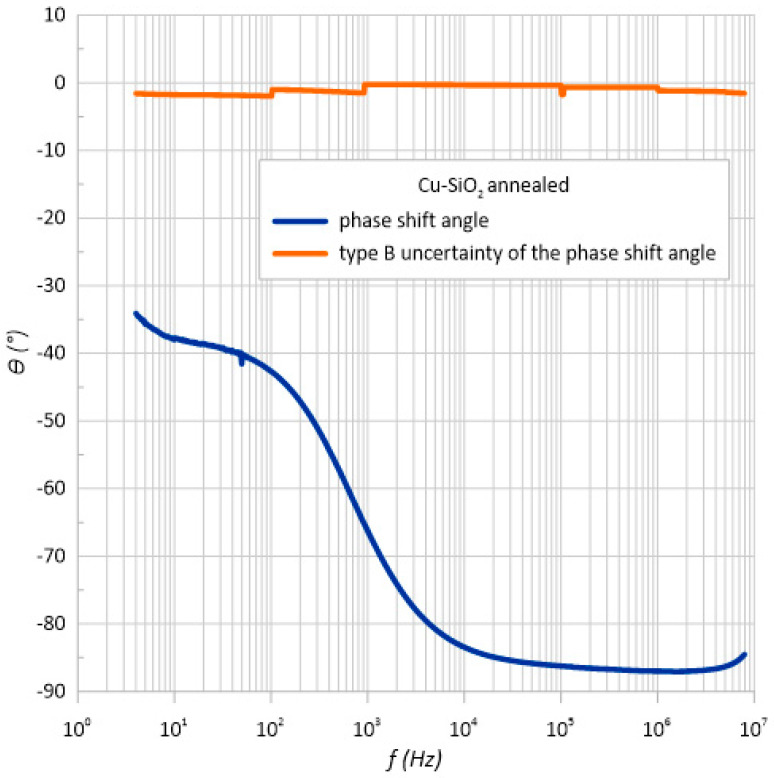
Frequency dependence of the impedance and standard impedance uncertainty of measurement type B *u*(*θ*) of the heated structure.

The meter’s specification states that it measures two electrical quantities such as the impedance and phase shift angle. Therefore, the measurement uncertainty of method B was also determined for the phase shift angle (Figure 14 and Figure 15). As can be seen, the measurement error varied to a maximum value of 3.75 degrees.

Table 3 presents the percentage values of errors determined by the *B* method for phase shift angle *θ* measurement points with different frequency values.

## 4. Conclusions

In this work, the technology of obtaining thin-film Cu–SiO_2_ nanocomposite structures by means of non-reactive magnetron sputtering was developed. A test stand was built to enable AC measurements in the frequency range from 4 Hz to 8 MHz and in the temperature range from room temperature to 393 K. Based on the obtained resistance results and mathematical calculations, the values of conductivity as a function of frequency were obtained, confirming the dielectric nature of the structure and the hopping conduction mechanism loads. The influence of annealing on the obtained granular nanocomposites was analyzed, and the equivalent diagram of the electric circuit was adjusted based on the phase shift angle diagram. Structural studies confirmed the granular character of the studied nanocomposite. Based on them, it was observed that the annealing process caused the oxidation of the copper particles, an increase in their diffusion barrier, and their merging into larger clusters. Additionally, an error analysis was carried out using the A method, which showed that for the unheated sample, the error was approximately 519 Ω, while for the heated sample, it was 119 Ω, which was 0.003 % and 0.0015 % of the measurement, respectively. However, in the case of the B-type standard uncertainty, based on the impedance measurements for the sample measured immediately after receipt, the error was very high. It is likely that the reasons for such high error values are the influence of electrical devices located in the same room, elements that make up the measuring station, and a very high resistance of the tested samples. In the case of measurements of the phase shift angle, such large values of measurement uncertainty were not observed.

## Figures and Tables

**Table 1:** List of measuring resistances and the value of the standard measurement uncertainty of type *A*.

Structure before Annealing						
*f,* Hz	$R_{p1}$	$R_{p2}$	$R_{p3}$	$R_{p4}$	Arithmetic Average, Ω	u_A_(R_p_), Ω
10	7.58 × 10^8^	7.52 × 10^8^	7.5 × 10^8^	7.5 × 10^8^	7.52 × 10^8^	1,594,641
100	5.64 × 10^8^	5.7 × 10^8^	5.69 × 10^8^	5.69 × 10^8^	5.68 × 10^8^	1,127,854
1 k	1.73 × 10^8^	1.73 × 10^8^	1.73 × 10^8^	1.73 × 10^8^	1.73 × 10^8^	30,671.73
10 k	18,278,700	18,273,400	1,8276,100	18,279,400	18,276,900	519.6152
100 k	1,829,310	1,829,550	1,829,860	1,829,340	1,829,515	59.1784
1 M	224,639	224,640	224,603	224,602	224,621	5.196152
**Structure after Annealing**						
10	91,705,800	92,375,600	90,983,000	92,493,300	91,889,425	53,007.97159
100	36,464,200	36,482,800	36,480,600	36,494,600	36,480,550	4719.838
1 k	18,457,000	18,455,300	18,457,800	18,458,600	18,457,175	50.51815
10 k	8,393,890	8,393,320	8,391,870	8,394,820	8,393,475	119.8002
100 k	1,606,470	1,607,910	1,606,910	1,607,400	1,607,172.5	202.7943
1 M	217,540	217,515	217,518	217,468	217,510.25	8.588085

**Table 2:** List of parameters necessary to determine the standard measurement uncertainty of type *B* for the measurements of impedance *Z*.

Structure before Annealing							
*f*, Hz	*Z*_1_, Ω	*Z*_2_, Ω	*Z*_3_, Ω	*Z*_4_, Ω	Arithmetic Average, Ω	*𝛿* (*Z*), %	*u*(*Z*), Ω
10	537,152,000	533,324,000	533,601,000	535,221,000	534,824,500	268.41225	82,880,6215
100	86,135,500	86,118,900	86,056,600	86,122,800	86,108,450	44.054225	21,901,442
1 k	9,202,700	9,202,420	9,203,060	9,203,300	9,202,870	3.159426	167,869.1331
10 k	984,202	984,199	984,174	984,169	984,186	3.152558	17,913.46671
100 k	107,014	107,015	107,016	107,012	107,014.25	0.50701425	313.257264
1 M	11,573.5	11,573.5	11,573.5	11,573.5	11,573.5	1.047205	69.9738542
**Structure after annealing**							
10	72,265,400	72,673,000	71,998,100	72,745,500	72,420,500	37.21025	15,558,347.93
100	26,817,300	26,827,600	26,825,800	26,826,400	26,824,275	14.4121375	2,232,008.079
1 k	7,434,060	7,433,290	7,433,320	7,434,160	7,433,707.5	3.5132585	150,783.8913
10 k	956387	956377	956383	956384	956,382.75	0.51308518	2833.091319
100 k	105671	105666	105669	105673	105,669.75	0.5989433	365.4060768
1 M	11,393.6	11,393.6	11,393.6	11,393.5	11,393.575	1.04180725	68.53095179

**Table 3:** List of parameters necessary to determine the standard measurement uncertainty of type *B* for measurements of phase shift angle *θ*.

Structure before Annealing							
*f*, Hz	*θ*_1_, °	*θ*_2_, °	*θ*_3_, °	*θ*_4_, °	Arithmetic Average, °	*𝛿* (*θ*), °	*u*_B_(*θ*), °
10	−44.872	−44.827	−44.621	−44.477	−44.6993	8.000013	−2.064573382
100	−81.219	−81.316	−81.294	−81.3	−81.2823	8.000024	−3.754277757
1 k	−86.949	−86.951	−86.952	−86.952	−86.951	4.000017	−2.00805606
10 k	−86.913	−86.913	−86.913	−86.914	−86.9133	0.708691	−0.355616972
100 k	−86.646	−86.647	−86.647	−86.646	−86.6465	0.708665	−0.354512185
1 M	−87.047	−87.047	−87.046	−87.046	−87.0465	1.326114	−0.666456111
**Structure after annealing**							
10	−38	−38.121	−37.69	−38.141	−37.988	8.000011	−1.754593062
100	−42.656	−42.663	−42.664	−42.686	−42.6673	8.000013	−1.970719015
1 k	−66.248	−66.248	−66.252	−66.25	−66.2495	0.600001	−0.229495504
10 k	−83.458	−83.457	−83.456	−83.458	−83.4573	0.600002	−0.289105198
100 k	−86.228	−86.232	−86.23	−86.231	−86.2303	0.700001	−0.348495836
1 M	−38	−38.121	−37.69	−38.141	−37.988	8.000011	−0.654273402

## Data Availability

Not applicable.
